# The Continuing Value of CD4 Cell Count Monitoring for Differential HIV Care and Surveillance

**DOI:** 10.2196/11136

**Published:** 2019-03-20

**Authors:** Brian Rice, Andrew Boulle, Sandra Schwarcz, Amir Shroufi, George Rutherford, James Hargreaves

**Affiliations:** 1 Faculty of Public Health and Policy Public Health, Environments and Society London School of Hygiene & Tropical Medicine London United Kingdom; 2 Faculty of Health Sciences Department of Public Health & Family Medicine University of Cape Town Cape Town South Africa; 3 Global Health Sciences University of California San Francisco San Francisco, CA United States; 4 Médecins Sans Frontières Cape Town South Africa

**Keywords:** CD4, HIV, differential care, antiretroviral therapy, surveillance, monitoring

## Abstract

The move toward universal provision of antiretroviral therapy and the expansion of HIV viral load monitoring call into question the ongoing value of CD4 cell count testing and monitoring. We highlight the role CD4 monitoring continues to have in guiding clinical decisions and measuring and evaluating the epidemiology of HIV. To end the HIV/AIDS epidemic, we require strategic information, which includes CD4 cell counts, to make informed clinical decisions and effectively monitor key surveillance indicators.

The CD4 cell count has been the principal basis for assessing an HIV-infected person’s level of immunosuppression and for timing initiation of antiretroviral therapy (ART) [1]. In 2015, the World Health Organization (WHO) recommended starting ART at any CD4 count, regardless of the clinical symptoms or conditions [2]. These guidelines and subsequent studies argue that, for clinical purposes, the frequency of CD4 monitoring post-ART initiation can be reduced or ceased when viral load testing is available and patients are suppressed [2-6].

The move toward universal ART, the expansion of viral load monitoring [7-9], and recommendations to reduce or cease CD4 testing post-ART initiation call into question the value of CD4 testing. Decreased support for CD4 testing could potentially not only result in reduced CD4 monitoring among people who have initiated ART, but also unintentionally lead to reduced quality of monitoring at diagnosis and the period up to ART initiation. In this communication, we highlight the continuing role of CD4 monitoring in guiding clinical decisions and measuring and evaluating the epidemiology of HIV.

The routine collection of CD4 data at diagnosis (baseline) from laboratories and health care facilities (conducting primary or confirmatory HIV tests) continues to provide an assessment of treatment priorities. Importantly, this information remains critical in identifying late diagnosis (as often indicated by a CD4 count of <350 cells/µL) [10-12]. A late diagnosis indicates that a person is at a significantly elevated risk of HIV-related opportunistic infections and mortality, and has been identified as a primary cause of HIV-related deaths in settings where ART is widely and freely available [10,12-13].

Low CD4 counts are triggers for more intensive follow-up and care in differentiated care models. For example, WHO guidelines on advanced disease recommend that people with a CD4 count <100 cells/µL be screened for cryptococcal disease and managed with fluconazole if asymptomatic, those with a CD4 <200 cells/µL receive tailored counselling, and those with a CD4 <350 cells/µL receive cotrimoxazole prophylaxis [14]. It is also recommended that people living with HIV with a count <100 cells/µL be offered the urinary lipoarabinomannan point-of-care test for tuberculosis [14]. In this way, CD4 is facilitating a shift away from symptom-based tuberculosis screening toward an approach of testing all those at high risk of disease. In Uganda, ART-naïve adults with CD4 counts ≤250 cells/µL are currently screened for tuberculosis and those with CD4 ≤100 cells/µL are also screened for cryptococcal antigen [15], similar to the case in South Africa [16]. In a recent three-country trial, presumptive antimicrobial treatment in patients initiating ART with CD4 counts <100 cells/µL resulted in a 30% reduction in 6-month mortality [17]. The routine collection and use of CD4 data have also been shown to be cost effective in promoting clinical outcomes such as disability-adjusted life years averted [18,19]. As loss to follow-up is a common outcome along the HIV care continuum [20], it is important that differentiated care models, informed by CD4 monitoring, also consider persons re-engaging in care.

Clinically mediated CD4 monitoring has also been an important feature of HIV surveillance. At the population level, the prevalence of CD4-defined late diagnoses helps monitor the effectiveness of program efforts for early identification [11,21] and is a WHO linkage to care indicator [21]. The linkage of CD4 data at diagnosis with longitudinal CD4 cell counts up to ART initiation has provided important information on trajectories of CD4 depletion between diagnosis and treatment. This information has been used at international, national, and subnational levels to back calculate from the time of diagnosis to the probable time of infection in order to estimate the incidence of HIV [22-26], estimate the prevalence of undiagnosed HIV [24-28], and assign a probable place of infection [29,30].

In a number of settings, the application of CD4-based models and their analyses are either being expanded or newly adopted. In 2016, a new model incorporating CD4 at or after diagnosis, but before ART, was introduced in the United States to estimate HIV incidence, prevalence, and undiagnosed infections [24]. Among European Union member states, a CD4 back-calculation model, which assigns probable place of HIV infection among migrant populations by estimating the time of infection and comparing it with the time of arrival in the host country, is being promoted to inform prevention programs [29,30]. In addition to informing pre-ART care and policy decisions concerning the use of ART for prevention [31], routine CD4 monitoring in South Africa has recently been used to assess the risk of subsequent loss to follow-up from care [32] and to estimate care cascade measures [33]. Although most of the CD4-based activities cited are focused in middle- and high-income settings, the promotion of HIV case surveillance [34] and the collection of CD4 within these systems will hopefully further expand the application of these methods to low- and middle-income settings, including high-prevalence settings in sub-Saharan Africa.

Clinical and surveillance activities relying on CD4 testing will be impaired if testing is reduced or discontinued between diagnosis and ART initiation, or in a setting where viral load testing remains suboptimal. Although it has been suggested that access to viral load monitoring in low-income, high-HIV burden settings may be limited [4], the Joint United Nations Programme on HIV/AIDS in 2016 reported that a number of resource-limited countries have drastically reduced CD4 monitoring in favor of increased viral load testing [35]. In 2018, the President’s Emergency Plan for AIDS Relief announced that they will reduce their overall level of support in donor countries for CD4 testing to prioritize viral load testing [36].

Although CD4 monitoring remains essential for the detection and management of HIV-related opportunistic infections such as *Cryptococcus*, signatories of a 2017 advanced HIV position statement claim donor support for CD4 testing at the primary care level has decreased in recent years [37]. The signatories of this statement included Médecins Sans Frontières. Figure 1 presents ART initiation by CD4 count in a Médecins Sans Frontières treatment program in South Africa. The top bar of the figure suggests an increase in recent years in the number and proportion of people not receiving a CD4 test at the time of treatment initiation. The reduction of CD4 monitoring both at and subsequent to diagnosis has also been brought to the attention of a research team carrying out HIV system assessments in resource-limited settings in 2015 and 2016 (R. Harklerode, personal communication, January 2018) [38].

While highlighting the role of continuing CD4 monitoring in informing clinical and epidemiological activities, we remain fully supportive of the expansion of viral load monitoring. In several areas of clinical management, for example, the monitoring of pediatric HIV infection [39], a combination of CD4 and viral load monitoring is essential. As CD4 tests are more affordable than viral load tests in many countries [18,19] and have already been scaled up, we believe CD4 monitoring presents a model of learning for scaling up optimal and affordable viral load testing.

It is inevitable that the role of CD4 monitoring in guiding clinical decisions will become more selective. However, vigilance and oversight are required to ensure that while we reduce reliance on CD4 monitoring in virologically suppressed patients, we retain our capacity to conduct CD4 testing at diagnosis and up to ART initiation. This remains critically important in diagnosing and treating comorbidities, determining whether a person requires an advanced package of screening and care, reducing mortality, and ensuing the continuity of critical data for surveillance activities (such as estimating HIV incidence and undiagnosed infections). Although we do not advocate for routine CD4 monitoring for all, CD4 should continue to guide the clinical management of persons re-engaging in care or remaining in care but failing treatment. Capacity will preferably be retained at the population level; where this is not the case, representative sampling methods should be considered. To end the HIV/AIDS epidemic, we must obtain essential data to make informed clinical decisions and effectively monitor key surveillance indicators.

**Figure 1:**
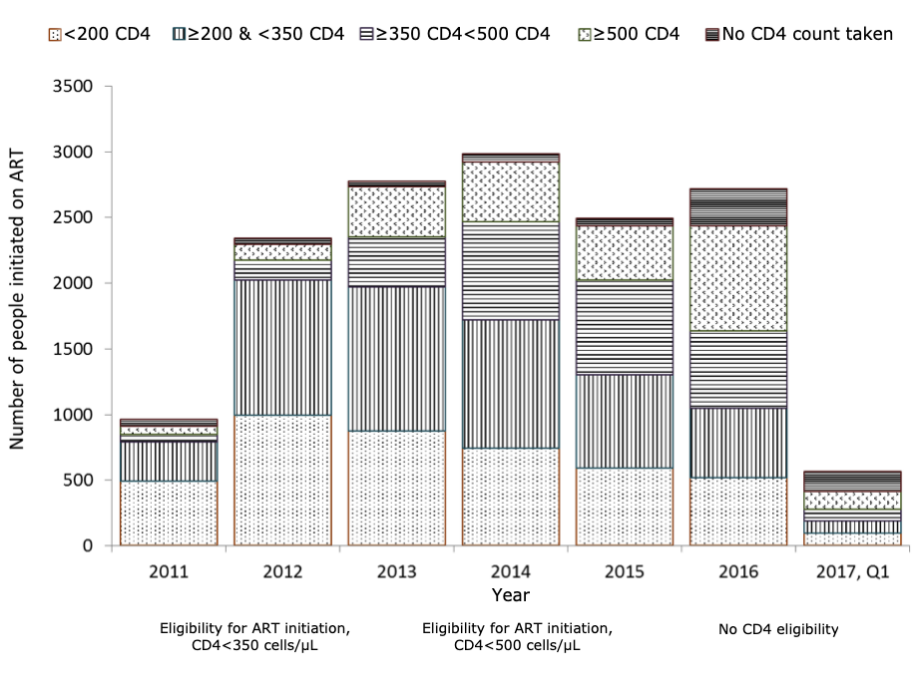
ART initiations in Eshowe and Mbongolwane, KwaZulu-Natal, stratified by CD4 count, 2011 to 2017. CD4 counts are measured as CD4 cells/µL. Q1 was from January to end March 2017. ART: antiretroviral therapy.

## Abbreviations

ART: antiretroviral therapy
WHO: World Health Organization
